# Is ^18^F-FDG PET/CT an Accurate Way to Detect Lymph Node Metastasis in Colorectal Cancer: A Systematic Review and Meta-Analysis

**DOI:** 10.1155/2020/5439378

**Published:** 2020-07-18

**Authors:** Hamid Dahmarde, Fateme Parooie, Morteza Salarzaei

**Affiliations:** ^1^Department of Radiology, Faculty of Medicine, Zahedan University of Medical Sciences, Zahedan, Iran; ^2^Students Research Committee, Zabol University of Medical Sciences, Zabol, Iran

## Abstract

**Aims:**

The purpose of this study was to assess the diagnostic value of ^18^F-fluorodeoxy-glucose positron emission tomography/computed tomography (FDG PET/CT) for detection of lymph node (LN) metastasis of colorectal cancer. *Material and Methods*. A computerized search was performed to determine the relevant articles, published before October 2019. Stata Statistical Software, version 15.0, and Meta-Disc (version 1.4) were used for the meta-analysis.

**Results:**

the sensitivity, specificity, positive likelihood ratio, and negative likelihood ratio were 0.65, 0.75, 4.57, and 0.37 respectively. Studies that used SUV_max_ cut-off value (≤2.5) demonstrated the best accuracy.

**Conclusion:**

^18^F-FDG PET/CT shows a low sensitivity and high specificity for detecting the metastasis of LNs in patients with newly diagnosed colorectal cancer.

## 1. Introduction

Worldwide, colorectal cancer (CRC) is the second most common cancer in women and the third in men [1]. CRC is the second leading cause of cancer deaths in the United States and according to estimates, 135,430 new cases and 50,260 cases of CRC deaths have occurred in 2017 [2]. Surgery and chemotherapy are two main ways to treat CRC. However, treatment outcomes for CRC have not been satisfactory due to high recurrence rate and metastasis, especially in patients with advanced CRC [3–6].

Lymph node (LN) metastasis is one of the most important prognostic factors for patients with CRC [3]. In addition, survival is directly related to the presence of residual metastatic LNs after primary surgery. Accurate diagnosis of LN metastasis at early stage may improve diagnosis and prompt the initiation of second-line treatment in patients with rectal cancer [4]. Conventional imaging techniques play an important role in the detection of malignant lymph node metastases [7, 8].

The goals of preoperative imaging are to determine the size of the primary tumor, adjacent organs involved, distant metastases or concurrent malignancy. This allows for a more accurate staging, which is of the utmost importance in planning the treatment. However, these methods only reflect the size, density, and morphology of lymph nodes: the biological activity and invasiveness of lymph nodes cannot be determined by conventional imaging techniques. Therefore, alternative imaging modalities that better reflect the biological behavior of LNs in CRC are of great importance.

Several methods have been developed to modify the partial volume effect and have significantly improved the diagnostic accuracy of metastatic LNs [9, 10]. However, there have been several limitations in the clinical use of partial volume correction due to the complexity of the procedure. Conventional computed tomography (CT) and magnetic resonance imaging (MRI) have been commonly used to stage LN in patients with rectal cancer, and lymph nodes larger than 2 cm have to be identified by standard CT scan. However, both CT and MRI are limited by the low sensitivity in the evaluation of small metastatic LNs [5–11].

Recently, it has been proven that F-18 fluorodeoxyglucose (FDG) positron emission tomography/computed tomography (PET/CT) can be useful for preoperative staging of rectal cancer by demonstrating metabolic information at the site of the lesion [12–14]. However, F-18 FDG PET/CT has shown low sensitivity for detecting LN metastasis [15, 16]. The low sensitivity of F-18 FDG PET/CT in the evaluation of metastatic LNs is mainly due to the partial volume effect that spills out the radioactivity into the background of small lesions <10 mm in size, which makes the actual standardized uptake value (SUV) insignificant. [17–19].

The purpose of this systematic review and meta-analysis was to assess the diagnostic value of F-18 fluorodeoxyglucose positron emission tomography/computed tomography (FDG PET/CT) for the detection of metastatic LNs of CRC.

## 2. Materials and Methods

### 2.1. Search Strategy

A computerized literature search to determine relevant articles, published before October 2019, was conducted by two authors (HD and FP) in PubMed, Google Scholar, and the Cochrane Library. The searched keyword combinations were as follows: colorectal cancer, lymph node metastasis, maximum standardized uptake value and “FDG” or “^18^F-FDG” and “PET” or “PET/CT.”

### 2.2. Inclusion and Exclusion Criteria

All potential articles were reviewed to determine whether they met the following criteria: (i) using PET/CT to evaluate metastatic LN characteristics; (ii) accurate determination of diagnostic criteria for malignant metastatic LNs or benign; (iii) explicit expression of sensitivity and specificity in the distinction between malignant metastatic and benign LNs; (iv) using the histopathologic results as the reference standard; (v) access to sufficient information to establish a 2 × 2 dependency table. Articles published in non-English languages, review articles, letters, comments, case reports, and articles which involved patients with known risk factors were excluded from the present analysis.

### 2.3. Data Extraction and Quality Assessment

Data from the desirable research were extracted independently by two reviewers (MS and FP), and any disagreements were resolved by a third author's judgment (HD). The data included the first author, country of study, year of publication, type of study (retrospective or prospective), number and gender of patients, diagnostic SUV threshold, and diagnostic results (TP, FP, FN, and TN). The methodological quality of each article was assessed by two reviewers (HD and MS) using the Quality Assessment of Diagnostic Accuracy Studies (QUADAS). The QUADAS tool consists of 14 items, each of which is rated as “high,” “low,” or “unclear” risk of bias, which were measured as “yes,” “no,” or “unclear” [15, 20].

### 2.4. Statistical Analysis

STATA statistical software, version 15.0 (Stata Corporation, College Station, Texas, USA), and Meta-Disc (version 1.4) were utilized for the meta-analysis. Based on the extracted information generated in the 2 × 2 dependency tables, sensitivity, specificity, positive likelihood ratio (PLR), negative likelihood ratio (NLR), and diagnostic odds ratio (DOR) were collected. All statistics were reported as point values with 95% confidence intervals (CIs). Sensitivity and specificity were calculated based on total positive/(total positive + false negative) and total negative/(total negative + false positive) formulas, respectively [16]. In addition, the area under the receiver operating characteristic curve (AUROC) was used as a global scale instead of the test performance. Also, a summary receiver operating characteristic (ROC) curve was created. A diagnostic test is considered complete when the AUROC is 100%. It was considered excellent, if AUROC is greater than 90%, and it was considered good, if AUROC is greater than 80%.

To assess the heterogeneity between the results of the articles, the *I*^2^ index and its *P* value were measured [21]. *P* < 0.05 or *I*^2^ greater than 50%, demonstrated the presence of heterogeneity. *I*^2^ ranged from 0 to 100, and the values of 0, 25, 50, and 75 showed no heterogeneity, low heterogeneity, moderate heterogeneity, and high heterogeneity between the results of the articles, respectively [22]. If there was a high heterogeneity, diagnostic performance was summarized using a random-effects coefficient binary regression model; otherwise, a fixed-effects coefficient binary regression model was used [23, 24]. Two methods were used to evaluate threshold effect performance using Meta-Disc (version 1.4) [25].

Using STATA 15.a, a strong positive correlation with *P* less than 0.05 between sensitivity and specificity would suggest the presence of a threshold effect. Given that heterogeneity could have been caused by other related factors, metaregression analysis and subgroup analysis were performed to investigate other potential factors that contributed to heterogeneity [26]. Metaregression analysis was performed by developing the Moses–Shapiro–Littenberg method [27]. A “*P*” less than 0.05 was considered significant. Subgroup analyses were performed in terms of type of study (prospective vs. retrospective), year of publication, reference standard (histopathology/follow-up vs. histopathology), and diagnostic threshold (cut-off value of SUV max ≥2.5 vs. >2.5). In addition, emission bias was assessed by two asymmetry and Deeks' funnel plot tests. An asymmetric funnel shape would indicate a significant bias. A regression of the logarithm of DOR (lnDOR) against half the effective sample size was used to calculate the degree of asymmetry. For the slope coefficient, *P* < 0.05 indicated a significant asymmetry of the funnel designs [28].

## 3. Results

### 3.1. Study Selection and Data Extraction

**Figure 1:**
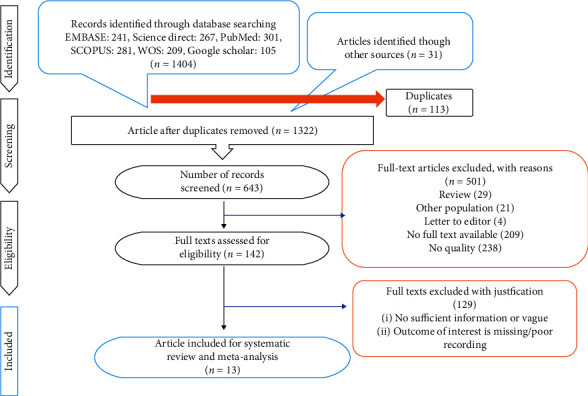
PRISMA flow diagram.

The initial computerized search discovered 1435 relevant articles. The titles and abstracts of relevant articles were reviewed by two reviewers. 142 full-text articles were selected for review. After screening the 142 full-text articles, we excluded 129 relevant articles for the following reasons: (i) studies which only included cancer staging; (ii) studies which selected disease population; (iii) insufficient data to construct a 2 × 2 table. Two additional studies were selected through reference papers. Finally, 13 eligible studies were included in the present meta-analysis. The process of selection of studies in the meta-analysis is presented in Figure 1.

### 3.2. Study Characteristics

Table 1 demonstrates principal characteristics of the 13 eligible studies. Data from the 13 articles included a total of 1460 patients, median age 58 years (range: 23–89 years). All patients of included studies had lymph nodes of 3 cm or less in diameter (range: 1–30 mm). The definite nature of LNs was affirmed on the basis of histopathological findings or a combination of radiological follow-up. Five studies were prospective and eight studies were retrospective.

### 3.3. Quality Assessment

**Figure 2:**
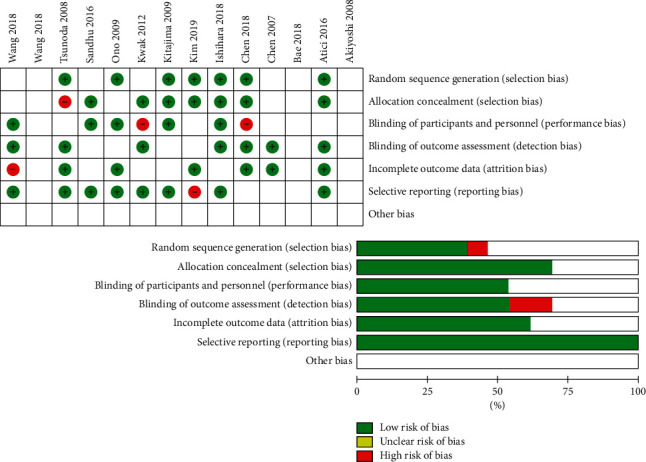
The risk of bias in the studies conducted was measured by using QUADAS-2 tool. The risk of bias demonstrated in equation 2 of each diagram shows the number and percentage of studies with high (red), medium (yellow), and low (green) risk of bias in four groups of QUADAS-2 tool.

The evaluation findings of QUADAS-2 are shown in Figure 2. The findings indicate that there is a risk of bias of evaluation for one parameter. These studies have certain limitations (Figure 2).

### 3.4. Diagnostic Performance

**Figure 3:**
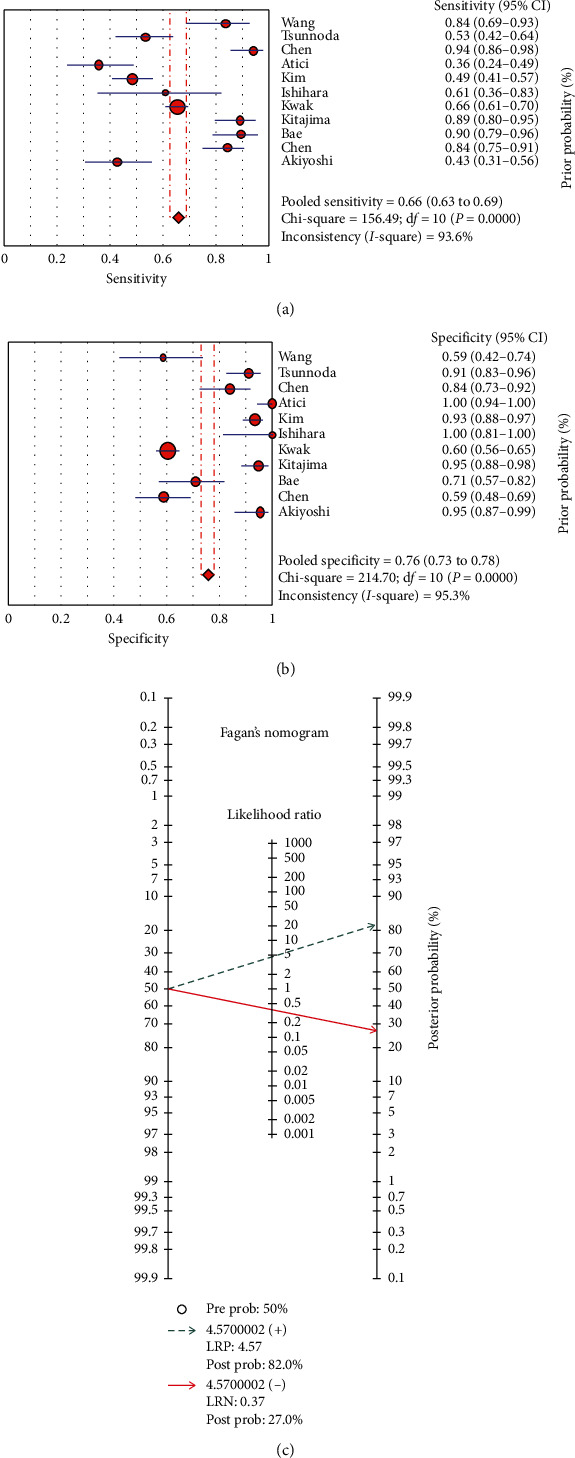
Sensitivity and specificity of ^18^F-FDG PET/CT in diagnosis of lymph node metastasis in colorectal cancer. Forest plot of sensitivity reported in each study. Each study is identified by the name of the first author and year of publication, with circles representing individual study point estimates, size of each circle indicating relative contribution to data pooling (inverse variance weighting), horizontal lines indicating 95% CIs, and dashed vertical lines representing 95% CIs for pooled sensitivity and specificity. (a) Fagan's nomogram for the calculation of posttest probabilities. A pretest probability of 50% diagnostic tool was fixed, which was estimated by the number of symptomatic cases in selected studies. (b) ^18^F-FDG PET/CT had a posttest probability of 82%. The results were obtained by the following calculations: pretest odds = prevalence/1-prevalence; posttest odds = pretest odds × negative likelihood ratio (LR−) (LR+); posttest.

**Figure 4:**
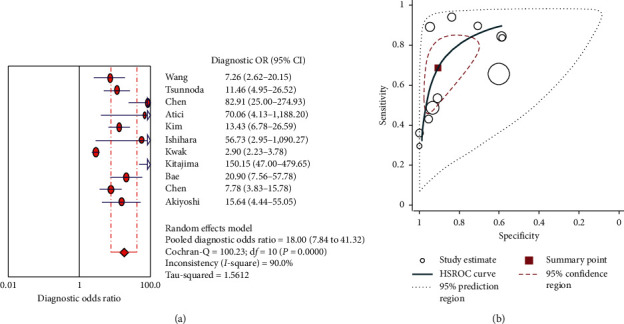
Diagnostic odds ratio for PET CT in diagnosis of lymph node metastasis in colorectal cancer (a) and hierarchical summary receiver (HSROC) curve for CEUS in diagnosis of lymph node metastasis in colorectal cancer (b).

Forest plots of the pooled sensitivity and specificity are demonstrated in Figure 3. A comparison between malignant and benign LNMs from 13 eligible studies [15, 18, 29–38] determined that the pooled sensitivity, specificity, positive likelihood ratio, and negative likelihood ratio with corresponding 95% confidence intervals (CI) were 0.65 (95% CI 0.63–0.68), 0.75 (95% CI 0.73–0.78), 4.57 (95% CI 2.84–7.35), and 0.37 (95% CI 0.28–0.48), respectively. The DOR with the corresponding 95% confidence intervals was 18.00 (95% CI 7.84–41.32) (Figure 4(a)). A summary HSROC curve was constructed and is depicted in Figure 4(b). The area under the SROC curve was 0.86, which showed good diagnostic accuracy. The heterogeneity test of sensitivities and specificities yielded *I*^2^ = 93.6% (*P* < 0.05) and *I*^2^ = 95.3% (*P* < 0.05), respectively. These results suggested notable heterogeneity among the studies included. The overall data were calculated using the DerSimonian Laird method on the basis of a random-effects model because of the presence of heterogeneity (*P* < 0.05 or *I*^2^ > 50%). The Spearman rank correlation coefficient was −0.31 (*P*=0.09) and our data showed no threshold effect. To investigate the possible sources of heterogeneity, we carried out a metaregression analysis using the extended Moses–Shapiro–Littenberg method. The results of metaregression analysis showed that the study design compared with other factors may be the most important source of heterogeneity (*P* < 0.05).

### 3.5. Subgroup Analyses

According to the findings of the metaregression analysis, we found that the study design was the most important source for heterogeneity. To further confirm the hypothesis, we carried out a subgroup analysis according to several variables of the studies included, including the study design, publication year, reference standard, and diagnostic threshold. The results of subgroup analysis are shown in Table 2. Prospective groups showed the best results for sensitivity 0.77 (95% CI 0.71–0.82). These groups with two reference standards had the highest specificity among the groups, 0.99 (95% CI 0.99–0.100). Prospective designed studies, studies with sample size <100, and those that used SUV_max_ cut-off value ≤2.5 demonstrated the best accuracy among the groups, 0.90 (95% CI 0.86–0.93), 0.90 (95% CI 0.87–0.94), and 0.92 (95% CI 0.90–0.95), respectively.

### 3.6. Meta-Analysis of Prevalence of Lymph Node Metastasis in Patients with Colorectal Cancer

**Figure 5:**
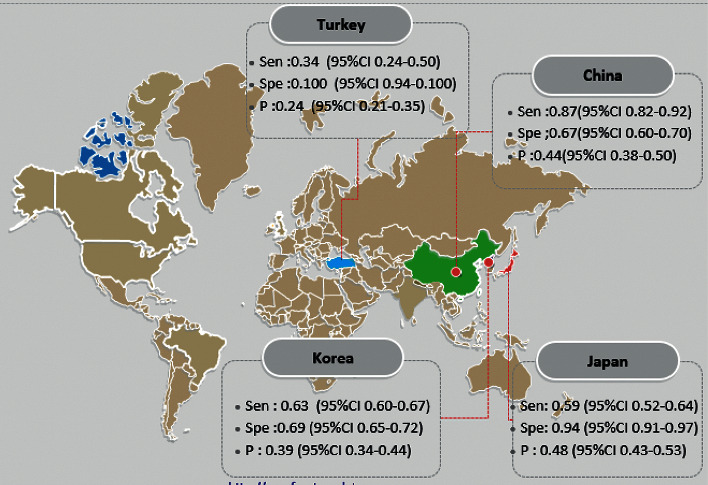
Meta-analysis of PET/CT in diagnosing lymph node metastasis in colorectal cancer based on country.

Based on the random effect model, the total prevalence of LN metastasis in 1460 patients with CRC was 40% (95% confidence interval [CI]: 37%, 43%, *I*^2^ = 65%) (Table 2; Figure 5).

### 3.7. Metaregression Finding Based on the Publication Year and Prevalence of Lymph Node Metastasis in Patients with Colorectal Cancer

**Figure 6:**
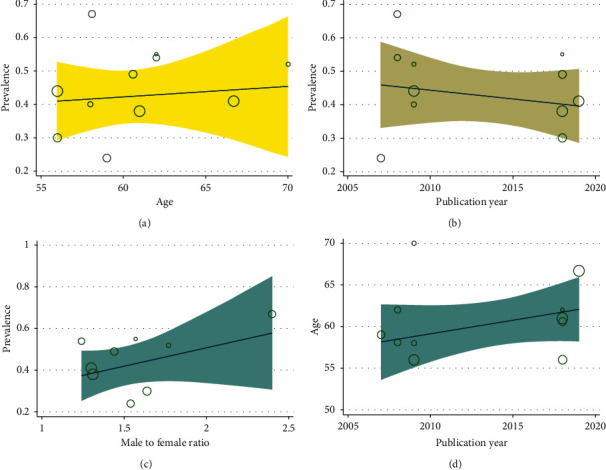
Metaregression between age (a), publication year of study (b), and male to female ratio (c) and accuracy of PET/CT in diagnosing lymph node metastasis in colorectal cancer based on country.

The studies' metaregression was according to the association between prevalence of LN metastasis and the publication year of study and the overall rate of LN metastasis. It showed the overall rate of LN metastasis was lower in newer studies than the older ones (Figure 6(b)). However, there was no statistically significant linear trend in univariate metaregression to explain effect size variation by publication year of study with coefficient = 9.50 (95% CI -25.58, 44.59); *P*=0.55 (Figure 6(b)).

### 3.8. Metaregression Finding Based on the Male to Female Ratio of Studies and Prevalence Lymph Node Metastasis in Patients with Colorectal Cancer

The overall rate of LN metastasis based on the male to female ratio of the study is shown in Figure 6(c). As shown in Figure 6(c), in the studies which had more male to female ratio, the rate of LN metastasis was higher. There was no statistically significant linear trend in univariate metaregression to explain effect size variation by male to female ratio of study with coefficient = 0.15 (95% CI −0.32, 0.63); *P*=0.42.

### 3.9. Metaregression Finding Based on the Patient Age and Publication Year of Studies of Lymph Node Metastasis in Patients with Colorectal Cancer

The studies' metaregression was according to the association between age and the publication year of studies. It demonstrated that the overall range of age was higher in newer studies than the older ones (Figure 6(a)). However, there was no statistically significant linear trend in univariate metaregression to explain effect size variation by publication year of study with coefficient = −158.76 (95% CI −1411, 1093.68); *P*=0.78 (Figure 6(a)).

## 4. Discussion

We evaluated the pretreatment function of ^18^F-FDG PET/CT as a staging modality to detect metastatic LNs in CRC. A previous meta-analysis investigating the role of ^18^F-FDG PET/CT in rectal cancer focused on detecting recurrent disease [39] or detecting metastasis in a population with early or recurrent cancer [4, 40]. The present meta-analysis provided a comprehensive overview of the literature, highlighted the causes of heterogeneity, and explored the clinical application of ^18^F-FDG PET/CT function in staging in this neoplasm in previous articles. ^18^F-FDG PET/CT was widely used for staging in a large number of oncologic diseases. For rectal cancer, this is used for staging tumors and LN, which may affect treatment planning.

Currently, the primary treatment for rectal cancer is external beam radiation and chemotherapy with 5-fluorouracil and mitomycin C [1]. The presence of metastatic LNs and their size will determine the amount of radiation for each LN [2]. The prediction of CRC is closely related to the histological type, invasion to the intestinal wall, malignant LN, type of surgery and recurrence after surgery, and/or metastasis. The emphasis on postoperative follow-up is due to local recurrence and distant metastasis, which are not detected by conventional imaging techniques such as MRI and ultrasound, until the lesion reaches a significant extent.

LN metastasis in rectal cancer is directly related to the disease prediction. The five-year survival coefficient is >95% in patients with CRC without LN metastasis but is reduced to 50–70% in patients with LN metastasis [3]. In addition, the LN stage of CRC is one of the most important determinants for adjuvant chemotherapy and LN dissection [4, 5]. Elective surgery for CRC patients with treatment stage N0 or N1 is total mesorectal excision, which is the excision of the mesorectal fat with all LNs.

In most advanced cancers with an N2 treatment stage, simultaneous chemotherapy and radiotherapy are recommended prior to surgery. Extensive LN dissection is required in patients with suspected metastatic LNs in the lateral pelvic region (6–8). The use of SUVmax cut-off values, optimized by LN size, improves the ability to determine treatment strategies and improves the prognosis of patients with CRC by improving the accuracy of LN metastasis detection using ^18^F-FDG PET/CT.

Although PET/CT imaging has pitfalls due to high FDG uptake by physiological causes, for example, increased FDG uptake due to inflammation, benign thyroid nodules, recent chemoradiotherapy, unilateral cranial nerve palsy, and recent surgery, this diagnostic tool provides a whole-body overview of a test and can detect abnormal glucose metabolism before morphologic changes of a lesion can be identified. As a result, this technique has become an effective and accurate noninvasive test in the follow-up of CRC surgery [9].

In this meta-analysis, a total of 13 major articles were included. As each article has a limited number of topics, this meta-analysis was conducted to integrate more information and provide more valid results. A number of meta-analyzes have been published on LN metastasis in CRC previously; however, our meta-analysis is the first meta-analysis to evaluate the diagnostic performance of PET/CT to detect LN metastasis in patients with CRC. Also, the quality of our research methodology using the accredited QUADAS-2 tool was tested. The data was substantially reviewed and meta-analysis of data from eight retrospective and five prospective studies determined that FDG-PET is a diagnostic tool with high specificity but is less sensitive at detecting locoregional LN involvement in patients with rectal cancer. In accordance with our data analysis, the prevalence of LN metastasis in CRC was 40%. In their study, Naxerova et al. reported a 65% incidence for distant and LN metastases in CRC [41]. It was highlighted that the overall sensitivity and specificity of FDG-PET in the detection of LN metastases in CRC were 65% and 75%, respectively. Sensitivity was also higher in studies with a sample size greater than 100 patients (70% vs. 65%). The results of the present study also demonstrated higher sensitivity and specificity for prospective articles when compared with retrospective (89% vs. 69%) and prospective (76% vs. 66%) studies, respectively. Another meta-analysis also reported that the sensitivity, specificity, PLR, and NLR of ^18^F-FDG PET/CT in detecting pretreatment LN involvement in patients with CRC were 42.9 %, 87.9%, 28.2%, and 69%, respectively [42]. Heterogeneity between studies may be a potential source of bias. The present meta-analysis revealed heterogeneity in diagnostic sensitivity and specificity. This heterogeneity is probably due to differences in methodological aspects between different articles (Table 1). Baseline differences among patients in the included studies (Table 1) may also have contributed to the apparent heterogeneity of the results [43]. According to the multivariate metaregression analysis of the present study, no definitive variable was the source of the heterogeneity of the study. There were some limitations to this meta-analysis. A standard search of texts could identify only eight studies for evidenced synthesis. However, meta-analysis provides an overview of the currently available literature on the subject [44]. In addition, there was a considerable heterogeneity in the pooled analysis. The type of reference standard used and the use of histological confirmation may explain the heterogeneity. None of the included studies provided a lesion-based analysis because a head-to-head comparison of surgical and PET/CT findings is impractical.

## 5. Conclusion

^18^F-FDG PET/CT demonstrates a low sensitivity and high specificity for detecting the metastasis of LNs in patients with newly diagnosed CRC. Also, ^18^F-FDG PET/CT is only useful for the confirmation of LN metastasis (when positive) in patients with CRC.

## Figures and Tables

**Table 1:** Characteristics of the included studies.

Author	Year	Country	M/F	Study design	Study duration	Patient	Age mean ± SD or range
Bae et al. [18]	2018	Korea	100/76	Retro	2009–2016	176	—
Chen et al. [29]	2018	China	56/34	Retro	2011–2017	90	43–87
Akiyoshi et al. [30]	2008	Japan	36/29	Retro	2005–2007	65	37–64
Ono et al. [31]	2009	Japan	16/9	Retro	2004–2007	27	51–84
Sandhu et al. [32]	2016	UK	10/5	Pros		15	51.47 ± 13.53
Wang and Li [33]	2018	China	—	Retros	2015–2017	43	—
Tsunoda et al. [15]	2008	Japan	52/36	Pros	2004–2005	88	23–89
Atici et al. [34]	2016	Turkey	37/24	Pros	2008–2010	61	59.16 ± 11.3
Kim et al. [35]	2019	Korea	94/72	Retros	2009–2016	166	66.7 ± 10.6
Ishihara et al. [36]	2018	Japan	11/7	Retros	2012–2015	18	32–78
Kwak et al. [37]	2012	Korea	301/172	Retros	2004–2009	473	25–85
Chen et al. [38]	2007	China	28/20	Pros		68	27–77
Kijima et al. [12]	2009	Japan	—	Pros	2005–2008	170	35–81

**Table 2:** Meta-analysis of included studies.

Characteristic	No. of patients	No. of studies	SEN	SPE	PPV	NPV	ACC
Sample size							
<100	639	9	0.58 (95% CI 0.53–0.63)	0.83 (95% CI 0.79–0.87)	0.79	0.70	0.90 (95% CI 0.87–0.94)
≥100	821	4	0.66 (95% CI 0.62–0.69)	0.72 (95% CI 0.69–0.75)	0.70	0.68	0.87 (95% CI 84–90)
Study design							
Pros	402	5	0.77 (95% CI 0.71–0.82)	0.99 (95% CI 0.99–0.100)	0.89	0.76	0.90 (95% CI 0.86–0.93)
Retro	1058	8	0.64 (95% CI 0.61–0.67)	0.71 (95% CI 0.68–0.73)	0.69	0.66	0.89 (95% CI 0.86–0.93)
SUV_max_ cut off value							
≤2.5	965	9	0.64% (95% CI 0.59–0.69)	0.87% (95% CI 0.83–0.90)	0.73	0.64	0.92 (95% CI 0.90–0.95)
>2.5	498	4	0.65% (95% CI 0.62–0.68)	0.71% (95% CI 0.68–0.74)	0.73	0.71	0.77 (95% CI 0.72–0.82)
All							
—	1460	13	0.65 (95% CI 0.63–0.68)	0.75 (95% CI 0.73–0.78)	0.73	0.69	89% (95% CI 0.86–0.91)

## Data Availability

The data used to support the findings of this study are available from the corresponding author upon request.

## Additional Points

(i) The prevalence of lymph node metastasis in colorectal cancer was 40%.The overall sensitivity and specificity of FDG-PET in the detection of lymph node metastases in colorectal cancer were 65% and 75%, respectively. (ii) PET/CT imaging has pitfalls due to high FDG uptake by physiological causes, increased FDG uptake due to inflammation, benign thyroid nodules, recent chemoradiotherapy, unilateral cranial nerve palsy, and recent surgery. ^18^F-FDG PET/CT is only useful for the confirmation of LN metastasis (when positive) in patients with colorectal cancer. (iii) Prospective designed studies, studies with sample size <100, and those that used SUV_max_ cut-off value ≤2.5demonstrated the best accuracy.
